# Unraveling the mysteries of macular telangiectasia 2: the intersection of philanthropy, multimodal imaging and molecular genetics. The 2022 founders lecture of the pan American vitreoretinal society

**DOI:** 10.1186/s40942-023-00505-5

**Published:** 2023-11-15

**Authors:** Lihteh Wu

**Affiliations:** 1 Asociados de Macula, Vitreo y Retina de Costa Rica, Primer Piso Torre Mercedes Paseo Colon, San Jose, Costa Rica; 2 Illinois Eye and Ear Infirmary, University of Illinois School of Medicine, Chicago, IL USA

**Keywords:** Macular telangiectasia 2, MacTel2, Serine, 1-Deoxysphingolipids, MacTel project, Müller cell, Mitochondrial maculopathy

## Abstract

**Purpose:**

Offer a personal perspective on the scientific advances on macular telangiectasia type 2 (MacTel2) since the launch of the MacTel Project in 2005.

**Design:**

Literature review and personal perspective.

**Methods:**

Critical review of the peer-reviewed literature and personal perspective.

**Results:**

Generous financial support from the Lowy Medical Research Institute laid the foundations of the MacTel Project. MacTel Project investigators used state of the art multimodal retinal imaging and advanced modern biological methods to unravel many of the mysteries surrounding MacTel2. Major accomplishments includes elucidation of the pathogenic role that low serine levels, elevated 1-deoxysphingolipids and other mechanisms induce mitochondrial dysfunction which lead to Müller cell and photoreceptor degeneration; the use of objective measures of retinal structures such as the area of ellipsoid zone disruption as an outcome measure in clinical trials; the demonstration that the ciliary neurotrophic factor slows down retinal degeneration and the development of a new severity scale classification based on multimodal imaging findings.

**Conclusions:**

MacTel2 is a predominantly metabolic disease characterized by defects in energy metabolism. Despite relatively good visual acuities, MacTel2 patients experience significant visual disability. The Mac Tel Project has been instrumental in advancing MacTel2 knowledge in the past two decades.

## Introduction

Retinal telangiectasias have been described in several conditions including diabetic retinopathy, retinal vein occlusions and radiation retinopathy among others. In 1982 Gass and Oyakawa [1] described a set of patients with no underlying conditions that manifested a bilateral juxtafoveolar telangiectasia in the temporal macular region. This was characterized by an age of onset between 40 and 60 years of age, graying of the parafoveal macula, intraretinal crystals, right angled vessels and late subretinal neovascularization (NV) in some patients. It is remarkable that regardless of the degree of damage, all the structural alterations and functional abnormalities are limited to an oval shaped area centered in the fovea known as the MacTel area. Furthermore the structural changes almost always begin in the temporal perifoveal area and spread circumferentially with progression of disease. The horizontal dimension of the MacTel area was defined as the distance between the foveal center and the temporal margin of the optic disc (approximately 3 mm) inclusive of the macula, fovea and the adjacent parafoveal areas. The vertical dimension is about 80% of this distance (approximately 2.5 mm) [2]. Currently this condition is named macular telangiectasia type 2 (MacTel2) and is considered a primary neurodegenerative condition with a secondary reactive vascular component.

Incidence and prevalence data are limited and probably underestimate the true values. Early disease is difficult to diagnose biomicroscopically since early signs and symptoms are non-specific and subtle. In addition late signs may be confused with age related macular degeneration (AMD). Several population based studies in different ethnic populations estimate the prevalence and incidence to be between 0.06 and 0.1%. However these are probably underestimated since these estimates are based solely on fundus photographs and not advanced multimodal imaging [3–5].

### The Mac Tel project

Developing treatments for rare conditions such as MacTel2 need to overcome several challenges. The most obvious hurdles include the high development and production costs of new drugs, long research and development timelines plus the competition of available resources from other more prevalent diseases. It goes without saying that a generous financial support plays an important role in advancing knowledge in each particular field [6]. Philanthropy through the Lowy Family Group (LFG) has been instrumental in financing the medical advancements in MacTel2. Over 15 years ago, an influential member of the LFG had a delayed diagnosis of MacTel2. The patient was surprised to learn that little was known about MacTel2. In addition he was told that there was no treatment and no ongoing research for this condition. The patient and his family decided to take matters into their own hands and fund research on MacTel2 [7]. The Lowy Medical Research Institute was established to fund the MacTel Project which was conceived in 2005. The MacTel Project represents an ongoing international collaboration between basic scientists and clinicians whose aim is to improve the understanding of MacTel2, to raise the awareness of the disease and to search for treatments. Their generous support has borne ample fruit as will be discussed.

### Functional changes

The MacTel Project conducted a large scale prospective natural history disease based on multimodal imaging. Thanks to the multimodal imaging findings of this study, a new severity scale for the progression of MacTel2 was recently developed and validated [8, 9]. This scale was based on the presence or absence and location (central vs non-central) of EZ disruption, SD-OCT hyper-reflectivity and pigment. The scale consists of 7 steps of increasing severity of visual acuity loss [9]. Severe vision loss is rare in MacTel2, only 4% of eyes and 0.7% of individuals were bilaterally affected with a BCVA ≤ 20/200. Photoreceptor atrophy was associated with severe vision loss [2]. Over an average follow-up of 4.2 years, visual acuity loss progressed very slowly at a rate of 1.07 letters per year [10]. However it must be borne in mind that traditional measures of visual function such as visual acuity do not reflect the full extent of visual dysfunction experienced by MacTel2 patients as exemplified by the results of a health related quality of life questionnaire [11]. Most patients present with binasal scotomas that lead to reading difficulties or visual distortion. There appears to be binocular inhibition of reading due to binocular rivalry. Affected left eyes may project scotomas in the reading direction [12].

Characterization of the functional deficits revealed that 43% of eyes had a unifocal absolute scotoma located in the temporal quadrant. After a mean follow-up of 5 years, the scotomas tended to progressively become more steeply sloped and grow but its growth never exceeded 40 deg [13]. Thanks to the development of a deep learning algorithm that creates maps of estimated retinal sensitivities from SD-OCT and microperimetric data, the disadvantages associated to microperimetry have possibly been overcome allowing for faster testing [14]. Dark adaptation is impaired in MacTel2. Rod-mediated recovery is more severely affected than cone-mediated recovery [15].

### Multimodal imaging, structural changes and clinicopathological correlation

In the original description of MacTel2, the vascular component was emphasized as noted in the name of idiopathic juxtafoveolar telangiectasis. Capillary telangiectasis, right angled venules and subretinal neovascularization featured in the Gass-Blodi staging of the disease, which was predominantly based on the vascular abnormalities observed on fluorescein angiography [16].

Advances in retinal multimodal imaging have implicated Müller cells in the pathogenesis of MacTel2 [17, 18]. SD-OCT findings include outer retinal hyper-reflectivity, intraretinal cavitations, pigmentary plaques and photoreceptor layer abnormalities [17]. The ellipsoid zone (EZ) integrity as imaged on en face OCT, which is considered a biomarker of photoreceptor health, is progressively lost albeit at a non-linear fashion [10, 19]. The rate of EZ loss is dependent on the size of the area of EZ loss at baseline [19]. For instance, 76% of eyes that did not manifest EZ loss at baseline developed EZ loss during the follow-up. In addition, 45% of eyes with established EZ loss at baseline had progression towards the center of the fovea. The extent of the loss of EZ integrity and its location with respect to the center of the fovea has been associated to loss of visual function [10]. The rates of visual acuity loss are similar in eyes with or without EZ loss unless the EZ loss involves the foveal center. Not surprisingly eyes with EZ loss that involved the foveal center had a significantly higher rate of visual loss of 1.40 letters per year [10]. There is also a strong topographic relationship between the telangiectatic vessels in the deep capillary plexus and the EZ loss seen on SD-OCT [20]. In eyes with early EZ loss, caution must be exercised when assessing the significance of the EZ loss. Not all EZ loss can be attributed to cone loss. A clinicopathological correlation of an eye with MacTel2 revealed the presence of cones in an area of EZ loss observed in the SD-OCT [21, 22]. In addition longitudinal studies have reported reductions in size and regressions of EZ loss in some eyes [23, 24]. Split detector adaptive optics scanning laser ophthalmoscopy (AO-SLO) but not other imaging modalities are able to detect the presence of remnant cone structures [22].

The intraretinal hyporeflective spaces observed in SD-OCT are not related to retinal thickening unlike retinal vascular conditions such as diabetic macula edema or cystoid macular edema secondary to retinal vein occlusions. They most likely represent photoreceptor loss if located in the outer retina whereas those in the inner retina probably occur secondary to a loss of Müller cells that are responsible for the structural integrity of the macula. Müller cell and photoreceptor cell loss lead to intraretinal cavitations.

OCT-A imaging has greatly expanded our understanding of the vascular abnormalities underlying MacTel2 [25–28]. The vascular density of both the superficial capillary plexus (SCP) and the deep capillary plexus (DCP) are decreased when compared to non-MacTel2 eyes. In mild disease the vessels in the DCP become dilated and telangiectatic, which rarely give rise to radial hemorrhages in Henle layer leading to a sudden loss of vision in affected individuals. Spontaneous resolution with recovery of vision confirms the absence of macular NV observed on OCT-A [29]. With more advanced disease, the vascular density decreases causing an absent or decrease flow in the DCP. Vascular invasion into the outer retina and subretinal space of these areas may be a compensatory mechanism to deal with the poor retinal perfusion [25]. In some eyes with photoreceptor loss, outer retina associated hyperreflectivity has been observed with SD-OCT. On OCT-A this outer retina associated hyperreflectivity represented NV. NV did not develop in eyes without photoreceptor loss [30, 31]. All eyes with NV had right angled vessels (RAV) that connected the inner retina to the deep neovascular complex [32]. RAV were defined by Gass and Blodi [16] as visible, blunted and slightly dilated vessels that dive at a right angle into the deeper retinal layers. These are usually located in the temporal parafoveal area and are considered to represent later stages of the disease process.

Intraretinal cavitations are not static and may change in shape, coalesce and collapse. Since these tend to occur in the temporal area, the asymmetric cavitation collapse will lead the remaining retina tissue to be pulled temporally and posteriorly [33]. The foveal avascular zone (FAZ) and the retinal vessels are dragged and pulled temporally and displaced posteriorly towards the RAV [26, 27]. The neuroretinal tissue descends towards the RPE bringing the DCP closer to the RPE plane. As the DCP approaches the RPE plane, connections between the deep capillary plexus and the choroidal circulation may form. In other eyes, collapse of the inner retinal layers brings both the DCP and SCP closer to the vitreoretinal interphase which may lead to epiretinal neovascularization [34].

Clinical pathological correlation of post-mortem eyes of patients with MacTel2 have confirmed the multimodal imaging findings [21, 35]. These eyes showed a loss of Müller cells in the MacTel zone and an absence of macular pigment that correlated topographically with the areas of Müller cell loss as suggested by multimodal imaging. In addition there is a subclinical phenotype characterized by subretinal debris, reduced outer segment phagosomes and ultrastructural RPE abnormalities not only in the MacTel area but throughout the central and peripheral retina [36, 37].

### Genetics, systemic lipid dysregulation and serine metabolism

In its original description MacTel2 was described as an acquired condition [1]. However multigenerational families have been described suggesting an autosomal dominant condition with variable penetrance and expressivity instead [38–44]. A recent study reported vertical transmission consistent with an incomplete penetrance autosomal dominant trait with a calculated genetic penetrance of 0.38 [42]. Causative genes for MacTel2 have been hard to identify given the reduced penetrance and the late onset of disease [43, 44]. Genome wide association studies (GWAS) identified 3 genetic susceptibility loci for MacTel2. These loci are associated with serine/glycine metabolism and vascular caliber [45]. A case control collapsing analysis based on whole exome sequencing revealed that functional variants in phosphoglycerate dehydrogenase (PHGDH), the rate limiting serine biosynthetic enzyme, accounts for 3.2% of MacTel2 cases [46]. Exome sequence analysis also identified gain of function variants in in the serine palmitoyltransferase long chain base subunit 1 (SPTLC1) and serine palmitoyltransferase long chain base subunit 2 (SPTLC2) genes as causative for both MacTel2 and hereditary sensory and autonomic neuropathy type 1 (HSAN-1), an extremely rare neurologic disease. An aberrant SPTLC1 or SPTLC2 accounts for < 0.1% of MacTel2 cases [47]. Patients with functional variants in PHGDH, SPTLC1 or SPTLC2 manifest low serum levels of serine and increased levels of 1-deoxysphingolipids [46, 47]. Metabolomic analysis of MacTel2 patients without any of these genetic variants also implicate low circulating serum levels of serine and increased levels of 1-deoxysphingolipids [45, 48].

Serine plays a central role in multiple metabolic pathways that regulate retinal homeostasis, particularly as a precursor of sphingolipid synthesis and free radical scavenging. Because of its high metabolic demand, the retina depends not only on the serine derived from the circulation but also on the de novo synthesis by the retinal glial cells and the RPE [49, 50]. When serine levels are low, serine palmitoyltransferase condenses palmitoyl-CoA with alanine instead of serine during sphingolipid synthesis giving rise to 1-deoxysphingolipids [47, 51]. Müller cells appear to be particularly sensitive to serine metabolic derangement. Investigators induced low levels of circulating serine in mice by feeding them a diet depleted of glycine and serine. These mice developed significant photopic and flicker electroretinographic deficits. 1-deoxysphingolipids have been shown to cause photoreceptor cell death in retinal organoids [47]. At a cellular level, reduction in serine production and 1-deoxysphingolipid accumulation both lead to mitochondrial disruption [52, 53]. Electron microscopic connectomics techniques of an eye with MacTel2 demonstrated structural mitochondrial changes in all retinal layers [54]. Inhibition of de novo serine synthesis in an in vitro study of Müller cells demonstrated that normal mitochondrial function was hampered and led to Müller cell death particularly under conditions of oxidative stress [55]. Studies using induced pleuripotential stem cell (iPSC)-differentiated retinal pigment epithelial (iRPE) cells derived from patients with MacTel2 demonstrated that low serine levels were also present in these cells. Furthermore these iRPE cells also demonstrated mitochondrial dysfunction that was independent of reduced serine or elevated 1-deoxysphingolipid [50].

### Treatment

Recent results from a phase 2 clinical trial concluded that a surgical implant sutured to the pars plana that delivers ciliary neurotrophic factor (CNTF) via an encapsulated cell technology (ECT) slowed the progression of retinal degeneration in MacTel2 [56, 57]. In ECT, a semipermeable polymer encapsulates genetically engineered RPE cells. These cells secrete CNTF continuously at a steady rate into the vitreous cavity [56]. Structure function correlation has been assessed with microperimetry and SD-OCT. Structural changes such as the loss of the EZ integrity preceded microperimetric detection of retinal sensitivity loss [58]. Relative scotomas correlated with EZ disruption whereas absolute scotomas correlated with a loss of the outer nuclear layer. Unlike SD-OCT, microperimetry can be burdensome and requires long testing times and trained technicians. The performance of a fully automatic deep learning-based segmentation method, Deep OCT Atrophy Detection, against the conventional semiautomatic measurements by expert readers method has been validated [59]. The fully automatic segmentation algorithm compared favorably to expert human readers when assessing the EZ defects on OCT images. Replacement of the current semiautomatic measurements by expert readers method by fully automatic segmentation may save money and time [59]. This clinical trial was one of the first to use images of retinal structure as a primary outcome measure of a clinical trial as a surrogate of visual function. These results provide evidence to support the use of objective measures of retinal structures such as the area of EZ disruption as an outcome measure. Future trials may benefit from this design since relevant outcomes can be reached earlier as compared to traditional measures such as visual acuity or visual field sensitivity [57, 60].

### Future directions

MacTel2 is a genetically complex disease. Although several genetic causes have been described the vast majority of genetic causes of MacTel2 remain unknown. Potential candidates include the *CHCHD2* gene, a mitochondrial regulator, and the *CYP2U1* gene which plays a role in the electron trnasport chain. Mitochondrial GWAS may also shed some light in this respect [50].

Once treatments become available, early detection of asymptomatic individuals through multimodal imaging screening will be important so that they may be prophylactically treated with neuroprotective drugs such as CNTF. Other potential treatments include fenofibrate, replacement gene therapy or serine supplement therapy. Fenofibrate is a peroxisome proliferator-activated receptor α (PPAR-α) agonist that has been shown to stimulate the degradation of deoxysphingolipids, to significantly decrease deoxysphingolipid induced photoreceptor cell death in retinal organoids and to lower the plasma levels of deoxysphingolipids in dyslipidemic patients [47]. The discovery of the defects in energy metabolism open different doors for potential treatments.

After almost 20 years, the MacTel Project has accomplished all the goals it initially set itself to do. Its major achievements includes elucidation of the pathogenic role that serine and 1-deoxysphingolipid metabolism abnormalities play in this condition; the use of objective measures of retinal structures such as the area of ellipsoid zone disruption as an outcome measure in a clinical trial [56, 60]; the demonstration that CNTF slows down retinal degeneration [56]; and the development of a new classification system based on multimodal imaging [9]. In conclusion the MacTel Project has played an important role in deciphering this puzzle called Mac Tel 2.

## Data Availability

Not applicable.
